# Vaccine Enthusiasm and Hesitancy in Cancer Patients and the Impact of a Webinar

**DOI:** 10.3390/healthcare9030351

**Published:** 2021-03-19

**Authors:** Amar H. Kelkar, Jodian A. Blake, Kartikeya Cherabuddi, Hailee Cornett, Bobbie L. McKee, Christopher R. Cogle

**Affiliations:** 1 Division of Hematology and Oncology, Department of Medicine, College of Medicine, University of Florida, Gainesville, FL 32610, USA; amarhkelkar@gmail.com; 2 Office of Community Outreach, Engagement & Equity, UF Health Cancer Center, Gainesville, FL 32610, USA; blake.j@ufl.edu; 3 Division of Infectious Disease & Global Medicine, Department of Medicine, College of Medicine, University of Florida, Gainesville, FL 32610, USA; kartikeya.cherabuddi@medicine.ufl.edu; 4 North Central Florida Cancer Control Collaborative Consortium, Well Florida Council, Gainesville, FL 32606, USA; hcornett@wellflorida.org; 5 Florida Cancer Control and Research Advisory Council, H. Lee Moffitt Cancer Center, Tampa, FL 33612, USA; bobbie.mckee@moffitt.org

**Keywords:** COVID-19, SARS-CoV2, vaccine hesitancy, vaccine, public health, patient education

## Abstract

(1) Background: Vaccine hesitancy and rejection are major threats to controlling coronavirus disease 2019 (COVID-19). There is a paucity of information about the attitudes of cancer patients towards vaccinations and the role of clinical oncologists in influencing vaccination acceptance. (2) Methods: Cancer patients and caregivers were invited to participate in a webinar and two surveys (pre- and post-webinar) assessing intention and thought processes associated with receiving COVID-19 vaccines. (3) Results: Two hundred and sixty-four participants participated in the webinar and registered to take at least one survey. Participants reported receiving most of their COVID-19 vaccine information from their doctor, clinic, or hospital. Before the webinar, 71% of participants reported the intention to receive a COVID-19 vaccine, 24% were unsure, and 5% had no intention of receiving a vaccine. The strongest predictors of vaccine enthusiasm were (a) planning to encourage the vaccination of family, friends, co-workers, and community, and (b) physician recommendation. The chief reason for vaccine hesitancy was a fear of side effects. After the webinar, 82.5% reported the intention to receive a vaccine, 15.4% were still unsure, and 2% stated that they had no intention of receiving a vaccine. The webinar shifted the attitude towards vaccine enthusiasm, despite an already vaccine-enthusiastic population. Communicating about vaccines using positive framing is associated with greater vaccine enthusiasm. (4) Conclusions: Patient education programs co-hosted by multiple stakeholders and delivered by oncologists can increase cancer patient enthusiasm for COVID-19 vaccination.

## 1. Introduction

In the historic process of rapidly developing, testing, and approving coronavirus disease 2019 (COVID-19) vaccines for emergency use authorization, certain populations have yet to be fully studied, including cancer patients [1,2]. However, the pressing threat of COVID-19 in cancer patients has supported a broad recommendation for cancer patients to receive vaccinations against COVID-19, with some specific considerations [3,4,5]. In deploying COVID-19 vaccines, the medical community and government agencies are confronting vaccine hesitancy and the potential for slow vaccine uptake [6,7,8].

Prior to COVID-19, vaccine hesitancy was a mounting problem in the US and worldwide. In the early months of COVID-19, there were signs of vaccine hesitancy worsening. As few as 50% of people surveyed reported the intention to receive a vaccine, which were still in testing at that point [7,9]. However, more recent surveys have shown declining vaccine hesitancy [10,11]. This change in sentiment may have been due to the combination of winter peaks in COVID-19 cases and case fatalities, as well as tangible vaccine emergency authorization. Nonetheless, there remain significant concerns that society will not reach adequate vaccination levels to achieve community immunity [12].

Although vaccine decision-making has been studied among parents, the general public, and health care professionals, there are no studies of vaccine decision-making in cancer patients. Health care professional knowledge and attitudes towards vaccines are known to be important factors in the rate of influenza vaccine uptake [13,14,15]. This has been further reinforced in recent surveys pertaining to COVID-19 vaccines [9,11]. Therefore, as oncologists and cancer patient advocates, we created and presented a webinar on COVID-19 vaccines for cancer patients and caregivers. We hypothesized that the webinar would increase knowledge about vaccinations against COVID-19 and change attitudes from vaccine rejection or hesitancy to vaccine acceptance or enthusiasm.

## 2. Materials and Methods

### 2.1. Study Design and Participants

Through the cooperation of the UF Health Cancer Center, the North Central Florida Regional Cancer Control Collaborative, and the Florida Cancer Control and Research Advisory Council (historically referred to as CCRAB), an educational webinar entitled “Cancer in the Time of Coronavirus: COVID-19 Vaccine” was developed using peer-reviewed literature, as well as public health information from the U.S. Centers for Disease Control and Prevention (CDC), U.S. Food and Drug Administration (FDA), World Health Organization (WHO), and New York Times. The webinar was advertised to members of the three co-hosting organizations via emails and social media posts (i.e., Facebook, Twitter, and Instagram). Webinar participation required pre-registration.

This study was approved by the University of Florida Institutional Review Board (IRB-02) for the use of a pre-webinar survey (Figure S1) and post-webinar survey assessing participant demographics, beliefs, and perspectives on vaccines and COVID-19. The pre-webinar survey was open from 31 December 2020 to the webinar date, which was 8 January 2021. The post-webinar survey was open from 8 January 2021 to 16 January 2021. Questions regarding vaccine beliefs and plans were designed based on the proposed causes of vaccine hesitancy and prior survey questions presented by the WHO and other studies [6,16,17].

### 2.2. Statistical Methods

The primary aims of the study were to observe endemic vaccine beliefs in a population of cancer patients and caregivers and determine which of these findings might be modifiable with community outreach and engagement. Secondary aims included determining the impact of age, gender, race, active treatment, political affiliation, and other key demographics on beliefs and vaccine plans. All statistical analyses were performed using IBM SPSS Statistics (Armonk, NY, USA). Descriptive statistics such as frequencies, means, and medians were used to analyze participant characteristics, sources of information, stated reasons for vaccine enthusiasm or hesitancy, and intentions to receive a vaccine. Automatic linear modeling for regression was used to create a predictive model of characteristics and beliefs associated with the choice to receive a COVID-19 vaccination. Ensemble modeling was used to boost the ability to identify predictors for vaccine enthusiasm or hesitancy. Paired *t*-tests were employed to compare pre- and post-webinar survey responses from the same individuals and *p* values < 0.05 were considered statistically significant.

## 3. Results

### 3.1. Participants and Characteristics

**Figure 1:**
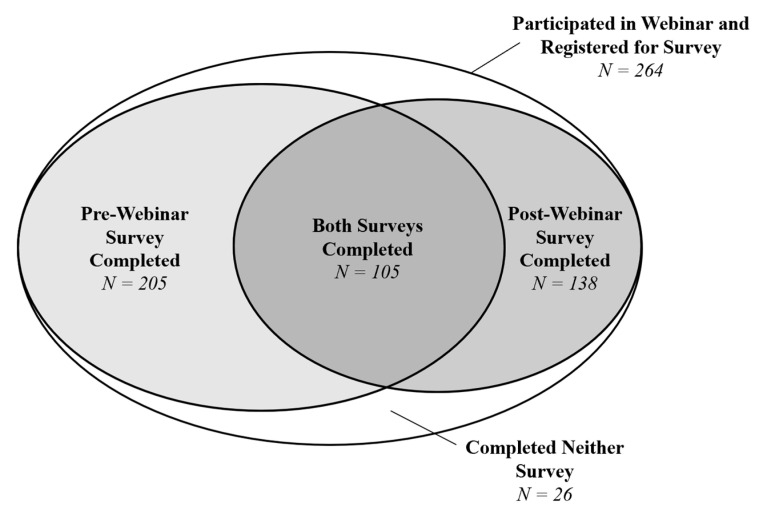
Number of participants who completed the pre-webinar and/or post-webinar surveys.

In total, 264 people participated in the webinar and registered to take at least one survey (Figure 1). Of these, 205 (78%) completed the pre-webinar survey, 138 (52%) completed the post-webinar survey, 105 (40%) people completed both the pre-webinar and post-webinar surveys, and 26 (10%) completed neither survey.

The characteristics of the three cohorts who completed the pre-webinar, post-webinar, or both surveys are described in Table 1. The characteristics among the cohorts were broadly similar; however, there was a higher percentage of people in the post-webinar cohort who preferred not to answer demographic questions. In general, respondents had personal connections to cancer and were older, White, female, educated, and affluent.

### 3.2. Where People Get Information about Vaccines against COVID-19

**Figure 2:**
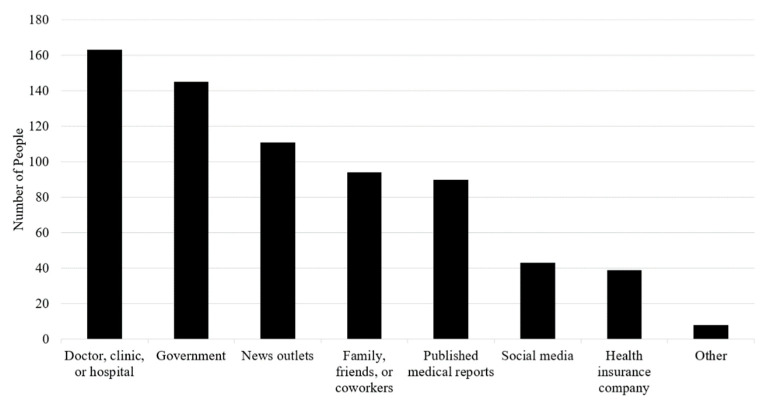
Sources of information about vaccines against coronavirus disease 2019 (COVID-19).

Participants reported a variety of sources of information about vaccines against COVID-19 (Figure 2). The most frequently cited source was a doctor, clinic, or hospital. Government agencies such as the CDC and the state Department of Health were the second-ranked source. News outlets were ranked next, followed by family, friends, or coworkers. Participants also reported receiving information from social media, such as Twitter and Facebook, and health insurance companies. Employers were mentioned as other sources of information about vaccines against COVID-19.

### 3.3. Characteristics Associated with the Intention to Receive a COVID-19 Vaccine

Before the webinar, 71% of people surveyed stated the intention to receive a COVID-19 vaccine, 24% were unsure, and 5% had no intention of receiving a vaccine.

The intention to receive a COVID-19 vaccine before the webinar (N = 205 participants) was analyzed in association with 31 variables, including participant demographics and beliefs about vaccines (Figure S1). Automatic linear modeling showed a prediction accuracy of 58% and identified two strong predictors of vaccine intention, both in the direction of vaccine enthusiasm (positive coefficient) (Table 2). There were no statistically significant predictors of vaccine hesitancy.

**Figure 3:**
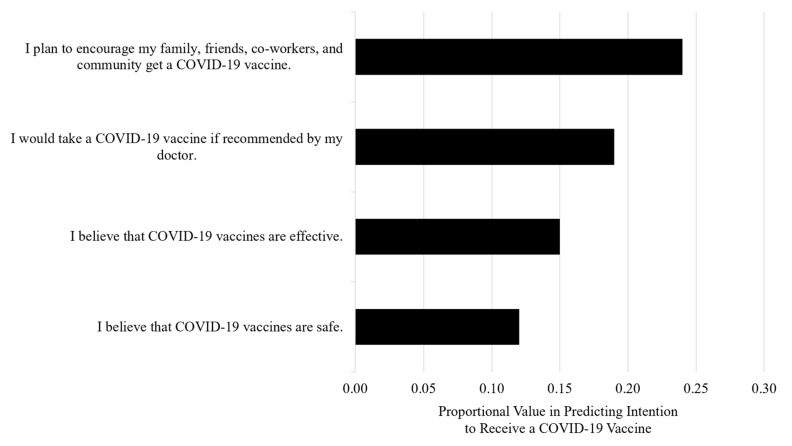
Predictors of the intention to receive a COVID-19 vaccine. Levels of model importance in predicting the intention to receive a COVID-19 vaccine based on webinar participant demographics and beliefs.

To boost the model accuracy, ensemble modeling was used for the same 31 demographic and belief variables from 205 participants. This iterative technique achieved a prediction accuracy of 59% and identified four variables with a higher predictive importance (Figure 3). The top two predictors were the same as predicted by the first linear regression model in Table 2, which confirmed their importance. The boosted model also identified beliefs in vaccine effectiveness and safety as important features associated with the intention to receive a COVID-19 vaccine.

### 3.4. Webinar Impact on the Intention to Receive a COVID-19 Vaccine

Reasons for vaccine hesitancy included concern about side effects from the vaccine (30%), a lack of information on COVID-19 vaccine effectiveness (14%), a lack of trust that the vaccine is safe for cancer patients (8%), a lack of information on where or how to get a COVID-19 vaccine (5%), a fear of needles (4%), a fear of contracting COVID-19 from the vaccine (2%), concern about payment for the vaccine (2%), disbelief that the COVID-19 vaccines are effective (1%), disbelief that the COVID-19 pandemic is significant (1%), and an allergy to other vaccines (1%). Less frequent reasons for vaccine hesitancy included concerns about vaccine interference with treatment for cancer, interference with fertility or pregnancy, preference to prevent with distancing and masking rather than vaccination, vaccine dilution because of a limited supply, a lack of long-term studies demonstrating safety, distrust of medical research because of historical racial discrimination, and an unsubstantiated claim by one participant that vaccines were made using cells from aborted fetuses.

To measure the extent by which the webinar changed the intention to receive a COVID-19 vaccine, responses from 97/105 individuals who answered the specific question about vaccine intention in both the pre- and post-webinar survey were studied. After the webinar, 80/97 (82.5%) of participants stated the intention to receive a COVID-19 vaccine, 15/97 (15.5%) were still unsure, and 2 (2.1%) stated no intention to receive a vaccine. The webinar increased the number of ‘yes’ respondents by 3, decreased the number of ‘maybe’ respondents by 2, and decreased the number of ‘no’ respondents by 1. Overall, the webinar shifted an already vaccine-enthusiastic population toward even greater vaccine enthusiasm and decreased vaccine hesitancy and rejection.

### 3.5. Changes in Beliefs and Perspectives on Vaccines against COVID-19

**Figure 4:**
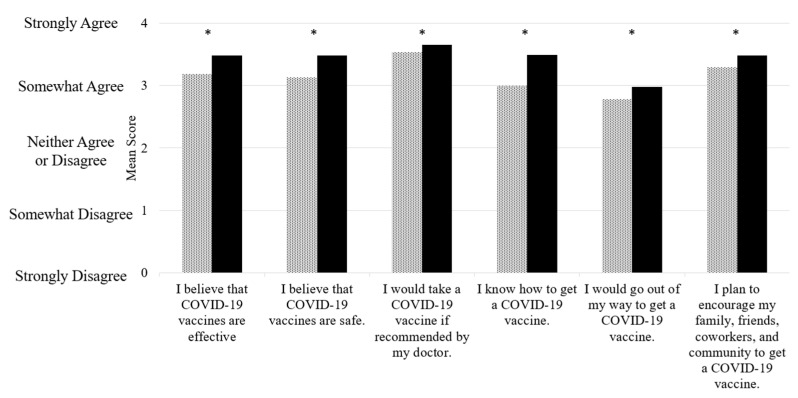
Changes in beliefs and perspectives on vaccines against COVID-19 before and after a webinar. Dotted bars represent data collected before the webinar. Black bars represent data collected after the webinars. A paired t-test used to compare before and after scores. *, *p* < 0.05.

Participants were queried before and after the webinar about their beliefs and perspectives on vaccines against COVID-19. In general, participants had favorable views of the vaccines (Figure 4). Despite high baseline agreeableness in relation to vaccines, the webinar further increased participants’ positive views of vaccines against COVID-19, specifically increasing agreeance with belief statements about vaccine effectiveness; vaccine safety; vaccine acceptance if recommended by a doctor; extra effort to receive a vaccine; and encouraging their family, friends, coworkers, and community to receive a vaccine.

### 3.6. Participants’ Understanding of the Vaccine Depends on Communication Framing

**Figure 5:**
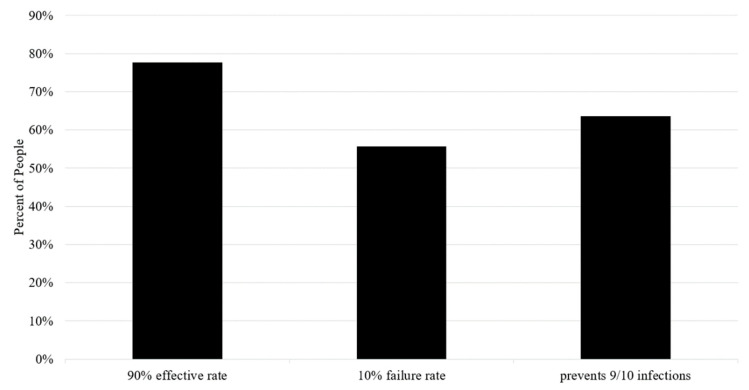
Which vaccine would you take?

When communicating the risks and benefits of vaccines, or any treatment, physicians must communicate probabilistic information. Clinicians can use different methods to communicate risks. In this study, three communication methods were tested to assess the association with vaccine enthusiasm or hesitancy. Specifically, participants were asked whether they would be willing to receive a vaccine with a 90% effective rate (positive framing), with a 10% failure rate (negative framing), or preventing 9 out of 10 people from being infected (frequency). The three answers are equivalent. However, more respondents (78%) chose to receive a vaccine worded with positive framing compared to a vaccine worded with frequency (64%) or negative framing (56%) (Figure 5).

## 4. Discussion

**Figure 6:**
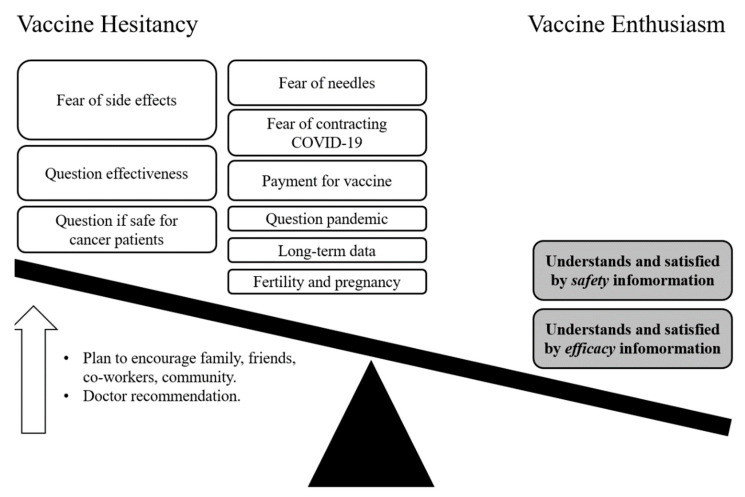
Model of vaccine hesitancy or enthusiasm in cancer patients. The size of the boxes is proportionate to their frequency in the cancer population participating in a webinar on vaccines and COVID-19. The gray boxes indicate the association between factors and the plan to receive a vaccine. The factors next to the up-arrow are associated with decreasing vaccine hesitancy in the cancer population participating in a webinar.

This study sheds light on vaccine acceptance among people with a history of cancer and their caregivers, which is a pressing issue in cancer centers and clinics worldwide. With limited time and resources, oncology clinicians, primary care providers, and patient advocacy organizations must choose how best to encourage COVID-19 vaccination for their patients with a history of cancer. Through the use of a webinar and surveys, this study exposed several mechanisms of vaccine hesitancy, but underscores two major points for physicians and patient advocates (Figure 6). First, physician authority is respected for COVID-19 vaccination. This factor was highly associated with positive attitudes toward receiving a COVID-19 vaccine. This finding has been previously reported for other vaccines, such as vaccines against influenza [13,14,15]. To shift public attitude toward vaccine acceptance, there need to be opportunities for physicians to directly and clearly engage with their patients and the public regarding vaccine safety and efficacy. Second, empathy gives purpose to COVID-19 vaccination. Study participants who were concerned about their family, friends, and coworkers were more likely to accept a vaccine for themselves. This worldview of human connection and shared sensibility speaks to the paradox of COVID-19 as both predator and prey to community action. Although messaging for COVID-19 vaccination has not yet incorporated empathy, the results from this study suggest that it should. One example might be “vaccinate if not for yourself, then for others.”

A further evaluation of patient attitudes found that vaccine enthusiasm was boosted by positive impressions of safety and efficacy data of the COVID-19 vaccines. In contrast, a fear of side effects was the chief concern of vaccine-hesitant participants. This study confirms and extends prior reports showing that the most formidable impediments to vaccine acceptance are safety concerns related to adverse effects and that allaying such concerns directly supports vaccine uptake [6,7,9,14,18,19]. This places an emphasis on high quality health communications and combating sources of misinformation. While our surveys were not designed to directly identify the role of misinformation in patient beliefs towards vaccines, some of these effects could be seen indirectly. The impact of social group influences is considered a major component of misinformation campaigns, through word of mouth and social media [20,21]. While seen to a lesser extent in our study, both these sources of information were used by many of our webinar attendees. We believe that these sources can be co-opted for the dissemination of high-quality information to promote safe vaccine usage; however, they remain threats in the campaign for vaccine acceptance [20]. This study supports the effort to create and deploy public-facing programs such as our webinar that serve to educate cancer patients about vaccinations [22,23].

This study also found that risk communication impacts attitudes toward COVID-19 vaccination. A higher number of participants elected to receive a vaccine when positive framing was used, compared to frequency wording or negative framing. Similar observations were observed when oncologists described the risks and benefits of chemotherapy for cancer [24]. This communication framing effect appears to hold for vaccines. When considering the relatively high educational status of our study participants, these findings raise concern for public susceptibility to negative framing by misinformed or nefarious agents or groups.

A few limitations should be considered when interpreting this study. First, the study population was predominately White, older, female, educated, and affluent, and had both access to and familiarity with videoconferencing and survey responses via the Internet. Of these factors, a White race, older age, higher educational attainment, and higher household income may have predisposed our study population to have a higher baseline level of vaccine enthusiasm [6,7,9]. At the baseline, this population expressed vaccine enthusiasm. Furthermore, some participants did not respond to demographic questions, which limited analyses. In the future, a longitudinal study design capturing the impact of the webinar on physically receiving a vaccine would also add value to the study results. Despite these limitations, the webinar still appeared to shift attitudes toward vaccine enthusiasm and away from vaccine rejection.

## 5. Conclusions

This study supports the use of cancer patient education programs about vaccinations where physicians clearly and directly present probabilistic data using positive framing and empathy. These results support the findings of prior studies exploring tools to combat vaccine hesitancy through community-level education. This effort also highlights the importance and opportunity for academic cancer centers, regional cancer collaboratives supported by the CDC and state departments of health, and state cancer councils to synergistically work together for cancer patient advocacy.

## Figures and Tables

**Table 1:** Characteristics of webinar participants who completed surveys. Three overlapping cohorts of people (238 unique individuals) completed surveys online before or after participating in a webinar on vaccines and coronavirus. A cohort of 205 people completed the pre-webinar survey, 105 people completed both surveys, and a cohort of 138 people completed the post-webinar survey. The characteristics of race, health insurance, and connections to cancer add up to more than 100% because respondents were allowed to choose more than one answer. For other characteristics, the total may not equal 100% because of rounding.

	Pre-Webinar Survey Completed (*N* = 205)		Both Surveys Completed (*N* = 105)		Post-Webinar Survey Completed (*N* = 138)	
**Characteristic**	**Number**	**Percent**	Number	Percent	Number	Percent
**Age (years)**						
18–26	10	5%	4	4%	4	3%
27–29	4	2%	0	0%	1	1%
30–39	20	10%	9	9%	9	7%
40–49	32	6%	14	13%	15	11%
50–59	35	17%	15	14%	15	11%
60–64	33	16%	20	19%	25	18%
65–69	19	9%	11	11%	1	1%
70–79	39	19%	23	22%	25	18%
80 years or older	11	5%	9	9%	11	8%
Prefer not to answer	2	1%	0	0%	32	23%
**Gender Identity**						
Man	40	20%	25	24%	27	20%
Woman	161	79%	78	74%	88	57%
Nonbinary, genderqueer, or genderfluid	1	0.5%	1	1%	0	0%
Prefer not to answer	3	2%	1	1%	23	17%
**Sexual Orientation**						
Heterosexual or “straight”	191	93%	97	92%	108	78%
Homosexual, gay, or lesbian	3	1.5%	2	2%	3	2%
Bisexual	2	1%	0	0%	1	1%
Other	1	0.5%	1	1%	1	1%
Prefer not to answer	8	4%	4	4%	25	18%
**Race**						
American Indian or Alaska Native	2	1%	0	0%	1	1%
Asian or Asian American	10	5%	3	3%	3	2%
Black or African American	12	6%	7	7%	8	6%
Native Hawaiian or Other Pacific Islander	1	0.5%	1	1%	1	1%
White	169	82%	90	86%	99	72%
Other	7	3%	2	2%	2	1
Prefer not to answer	6	3%	2	2%	24	17%
**Ethnicity**						
Hispanic or Latinx	18	9%	5	5%	5	4%
Not Hispanic or Latinx	175	85%	96	91%	107	78%
Prefer not to answer	12	6%	4	4%	26	19%
**Highest Level of Education**						
High school or equivalent	7	3%	3	3%	4	3%
Some college credits	15	7%	7	7%	9	7%
Associate’s degree	15	7%	7	7%	9	7%
Bachelor’s degree	60	29%	29	28%	32	23%
Graduate or professional degree	103	50%	56	53%	60	43%
Prefer not to answer	5	2%	3	3%	24	17%
**Economics**						
Number of people in household						
1	37	18%	24	23%	28	20%
2	106	52%	58	55%	65	47%
3	31	15%	10	10%	11	8%
4	17	8%	7	7%	8	6%
5	8	4%	2	2%	2	1%
6	3	1.5%	2	2%	2	1%
7	2	1%	2	2%	2	1%
8	0	0%	0	0%	0	0%
9 or more	1	0.5%	0	0%	0	0%
Prefer not to answer	0	0%	0	0%	20	14%
Total household income for 2020						
Less than $15,000	2	1%	1	1%	1	1%
$15,000 to $19,999	5	2%	3	3%	3	2%
$20,000 to $24,999	3	1.5%	1	1%	1	1%
$25,000 to $34,999	6	3%	4	4%	4	3%
$35,000 to $49,999	9	4%	8	8%	10	7%
$50,000 to $74,999	25	12%	12	11%	15	11%
$75,000 to $99,999	29	14%	15	14%	15	11%
$100,000 and above	71	35%	29	28%	32	23%
Prefer not to answer	55	27%	32	31%	57	41%
**Health Insurance**						
Employer offered	133	65%	59	56%	65	47%
Private purchase	37	18%	21	20%	26	19%
Medicare	67	33%	44	42%	49	36%
Veterans Affairs, Tricare	14	7%	7	7%	8	6%
Medicaid	6	3%	3	3%	3	2%
No health insurance	1	0.5%	0	0%	0	0%
Other (student health)	7	3%	3	3%	8	6%
Prefer not to answer	0	0%	0	0%	13	9%
**Political Affiliation**						
Democrat	69	34%	38	36%	47	34%
Republican	39	19%	19	18%	19	14%
Independent	29	14%	12	11%	14	10%
Other	4	2%	1	1%	2	1%
Prefer not to answer	64	31%	35	33%	56	41%
**Connections to Cancer**						
Have cancer and actively receiving treatment	55	27%	35	33%	40	29%
Cancer survivor and not receiving treatment	59	29%	24	24%	29	17%
Caregiver to a cancer patient	20	10%	12	11%	12	9%
Friend or family member to a cancer patient	47	23%	25	24%	27	20%
Health care provider	39	19%	15	14%	15	11%
Academic researcher or research staff member	18	9%	12	11%	14	10%
Government employee	8	4%	3	3%	3	2%
Community-based organization that serves people with cancer	20	10%	10	10%	10	7%
Health insurance company employee	1	0.5%	0	0%	0	0%
No connection to cancer	2	1%	0	0%	0	0%
Other (caregiver to person with other disease, for-profit business, family of health care worker)	12	6%	6	6%	8	6%

**Table 2:** Characteristics and beliefs associated with the intention to receive a COVID-19 vaccine. LINEAR modeling of 31 demographic and attitude variables in 205 people prior to participating in a webinar.

Characteristic	Coefficient	Significance	Model Importance
I plan to encourage my family, friends, co-workers, and community to get a COVID-19 vaccine.	0.260	*p* < 0.0001	0.548
I would take a COVID-19 vaccine if recommended by my doctor.	0.252	*p* < 0.0001	0.351

## Data Availability

The data presented in this study are available on request from the corresponding author. The data are not publicly available due to participant privacy.

## Supplementary Materials

The following are available online at https://www.mdpi.com/2227-9032/9/3/351/s1: Figure S1: Pre-webinar survey.
