# Supplementary material for: Vaccine Enthusiasm and Hesitancy in Cancer Patients and the Impact of a Webinar
Source: Healthcare (Basel). 2021 Mar 19;9(3):351. doi: 10.3390/healthcare9030351 (PMC8003419; doi:10.3390/healthcare9030351)
Supplement: Supplementary file 1 [file healthcare-09-00351-s001.pdf]

Consent

Consent to Be Surveyed

Dr. Chris Cogle and Dr. Amar Kelkar at the University of Florida would like your thoughts about vaccinations and the coronavirus. This study was approved by the University of Florida Institutional Review Board (IRB) under protocol IRB202003310. You must be over 18 years old or older to be surveyed. Although there is no personal benefit to participating in this study, the information will help doctors better understand the needs for community health education. There is no payment for your participation.

There will be two surveys: one survey before the webinar by Drs. Cogle and Kelkar, and a follow-up survey after the webinar. The email address that you provide in this first survey will be entered into a confidential directory so that a follow-up survey can be sent to you after the webinar. All response data will be studied anonymously and not specific to any particular email address. Your email address will be permanently deleted after the team receives your follow-up survey or one year after receiving your first survey results, whichever comes first. It is extremely unlikely that someone outside the research

team will access your email address because of the team's use of encryption and password protection. However, in that extremely unlikely event, if someone did illegally access your email address then they could use it to identify you, which could affect your employability or insurability (life insurance, long term care insurance). Again, this is extremely unlikely.

If you have any questions about this study, you may contact Dr. Cogle at (352) 273-7493. For information regarding your rights as a research participant contact the IRB Office at 352-392-0433.

I am 18 years old or older and give consent to be surveyed.

- ○ Yes
- ○ No

Email and Demographics

Please enter your email address:${q://QID102/ChoiceTextEntryValue}

Please confirm your email address:

What is your age?

- 18-26 years
- 27-29
- 30-39
- 40-49
- 50-59
- 60-64
- 65-69
- 70-79
- 80 years or older
- Prefer not to answer

What is your gender identity?

- Man
- Woman
- Transgender female or trans woman
- Transgender male or trans man
- Nonbinary, genderqueer, or genderfluid
- A gender not listed here

○ Prefer not to answer

Which of the following best describes how you think of yourself?

○ Heterosexual or "Straight"
○ Homosexual, Gay, or Lesbian
○ Bisexual
○ ________ Other
○ Prefer not to answer

What is your race? More than one may apply.

☐ American Indian or Alaska Native
☐ Asian or Asian-American
☐ Black or African-American
☐ Native Hawaiian or Other Pacific Islander
☐ White
☐ ________ Other
☐ Prefer not to answer

What is your ethnicity?

- Hispanic or Latinx
- Not Hispanic or Latinx
- Prefer not to answer

What is the highest level of school you finished?

- Less than high school
- Some high school
- High school or equivalent (for example, GED)
- Some college credits
- Associate's degree (for example, AA or AS)
- Bachelor's degree (for example, BA, BS, or AB)
- Graduate degree (for example, MA, MS, PhD, of professional degrees such as MSW, MD, DDS, DVM, PharmD, JD)
- Prefer not to answer

How many people live in your household?

- 1
- 2
- 3
- 4
- 5
- 6
- 7
- 8

○ 9 or more

In 2020, what was your total household income before taxes?

○ Less than $15,000
○ $15,000 to $19,999
○ $20,000 to $24,999
○ $25,000 to $34,999
○ $35,000 to $49,999
○ $50,000 to $74,999
○ $75,000 to $99,999
○ $100,000 and above
○ Prefer not to answer

How do you pay for your health care? Please select all that apply.

☐ Health insurance offered from my job or a family member's job
☐ Health insurance that I pay on my own
☐ Medicare
☐ Veterans Affairs, Tricare, Military
☐ Medicaid
☐ No health insurance
☐ [ ] Other

What is your political party affiliation?

- ○ Republican
- ○ Democrat
- ○ Independent
- ○ [ ] Other
- ○ Prefer not to answer

What is the ZIP code of your home residence?

[ ]

My connection to cancer include the following. (More than one answer may apply.)

- ☐ Have cancer and actively receiving treatment
- ☐ Cancer survivor and not receiving treatment
- ☐ Caregiver to a cancer patient
- ☐ Friend or family member of cancer patient
- ☐ Healthcare provider (for example, physician, nurse, social worker, etc)
- ☐ Academic researcher or research staff member
- ☐ Government employee (for example, Department of Health, AHCA, County government, City government)
- ☐ Community organization employee that serves people with cancer

- [ ] Health insurance company employee that serves people with cancer
- [ ] No connection to cancer
- [ ] ____________ Other

COVID-19 Vaccine

Do you plan on getting a vaccine for COVID-19?

- ○ Yes
- ○ Maybe
- ○ No

What are your concerns about COVID-19 vaccines, if any? Please select all that may apply.

- [ ] I have no concerns about getting a COVID-19 vaccine.
- [ ] I'm not concerned about getting sick from COVID-19.
- [ ] I don't believe the COVID-19 pandemic is as bad as some people say it is.
- [ ] I'm allergic to vaccines.
- [ ] I don't like needles.
- [ ] I'm concerned I might get COVID-19 from the vaccine.
- [ ] I'm concerned about side effects from the vaccine.
- [ ] I don't trust that the vaccine will be safe for people like me.
- [ ] I don't know enough about how well COVID-19 vaccines work.
- [ ] I don't think the COVID-19 vaccines work very well.

- [ ] I don’t want to pay for it.
- [ ] I don't know where or how to get a COVID-19 vaccine.
- [ ] Other

Please rate the following statements:

	Strongly agree	Somewhat agree	Neither agree nor disagree	Somewhat disagree	Strongly disagree
I believe that COVID-19 vaccines are effective.	○	○	○	○	○
I believe that COVID-19 vaccines are safe.	○	○	○	○	○
I would take a COVID-19 vaccine if recommended by my doctor.	○	○	○	○	○
I know how to get a COVID-19 vaccine.	○	○	○	○	○
I would go out of my way to get a COVID-19 vaccine, such as travel over 60 miles or wait for more than 60 minutes.	○	○	○	○	○
I plan to encourage my family, friends, co-workers, and community to get a COVID-19 vaccine.	○	○	○	○	○

Please rate the following statements:

	Strongly agree	Somewhat agree	Neither agree nor disagree	Somewhat disagree	Strongly disagree
I believe that COVID-19 vaccines contain microchips.	○	○	○	○	○
I believe that fifth generation (5G) broadband cell networks spread COVID-19.	○	○	○	○	○
I feel sad that over 350,000 people in the United States have died of COVID-19.	○	○	○	○	○
I feel afraid that something awful might happen.	○	○	○	○	○

Vaccines in General

Where do you get information about vaccines? More than one answer may apply.

- ☐ Family, friends, or co-workers
- ☐ Doctor, clinic, or hospital
- ☐ Social media (for example: Facebook, Twitter)
- ☐ Government (for example: Department of Health, CDC, NIH)
- ☐ News outlets (for example: radio, TV, newspaper, new websites)

- [ ] Health insurance company
- [ ] Published medical reports (for example: New England Journal of Medicine, JAMA)
- [ ] ____ Other - Please specify

Please rate the following statements:

	Strongly agree	Somewhat agree	Neither agree nor disagree	Somewhat disagree	Strongly disagree
I believe vaccines, in general, are effective at preventing infection.	○	○	○	○	○
I believe vaccines, in general, are safe.	○	○	○	○	○
I would take any medication recommended by my doctor.	○	○	○	○	○
I would take any vaccine recommended by my doctor.	○	○	○	○	○

Which of the following vaccines would you take? More than one answer may apply.

- [ ] A vaccine that is 90% effective at preventing infection.

- [ ] A vaccine that has a 10% failure rate at preventing infection.
- [ ] A vaccine that prevents infection in 9 out of 10 people.

Powered by Qualtrics
